# Complexity in psychological self-ratings: implications for research and practice

**DOI:** 10.1186/s12916-020-01727-2

**Published:** 2020-10-08

**Authors:** Merlijn Olthof, Fred Hasselman, Anna Lichtwarck-Aschoff

**Affiliations:** 1 Behavioural Science Institute, Radboud University, Nijmegen, The Netherlands; 2 School of Pedagogical and Educational Sciences, Radboud University, Nijmegen, The Netherlands

**Keywords:** Ecological momentary assessment, Experience sampling method, Complexity, Complex system, Psychopathology, Mental health, Time series, Personalized medicine

## Abstract

**Background:**

Psychopathology research is changing focus from group-based “disease models” to a personalized approach inspired by complex systems theories. This approach, which has already produced novel and valuable insights into the complex nature of psychopathology, often relies on repeated self-ratings of individual patients. So far, it has been unknown whether such self-ratings, the presumed observables of the individual patient as a complex system, actually display *complex* dynamics. We examine this basic assumption of a complex systems approach to psychopathology by testing repeated self-ratings for three markers of complexity: *memory*, the presence of (time-varying) short- and long-range temporal correlations; *regime shifts*, transitions between different dynamic regimes; and *sensitive dependence on initial conditions*, also known as the “butterfly effect,” the divergence of initially similar trajectories.

**Methods:**

We analyzed repeated self-ratings (1476 time points) from a single patient for the three markers of complexity using Bartels rank test, (partial) autocorrelation functions, time-varying autoregression, a non-stationarity test, change point analysis, and the Sugihara-May algorithm.

**Results:**

Self-ratings concerning psychological states (e.g., the item “I feel down”) exhibited all complexity markers: time-varying short- and long-term memory, multiple regime shifts, and sensitive dependence on initial conditions. Unexpectedly, self-ratings concerning physical sensations (e.g., the item “I am hungry”) exhibited less complex dynamics and their behavior was more similar to random variables.

**Conclusions:**

Psychological self-ratings display complex dynamics. The presence of complexity in repeated self-ratings means that we have to acknowledge that (1) repeated self-ratings yield a complex pattern of data and not a set of (nearly) independent data points, (2) humans are “moving targets” whose self-ratings display non-stationary change processes including regime shifts, and (3) long-term prediction of individual trajectories may be fundamentally impossible. These findings point to a limitation of popular statistical time series models whose assumptions are violated by the presence of these complexity markers. We conclude that a complex systems approach to mental health should appreciate complexity as a fundamental aspect of psychopathology research by adopting the models and methods of complexity science. Promising first steps in this direction, such as research on real-time process monitoring, short-term prediction, and just-in-time interventions, are discussed.

## Background

Complex systems approaches to mental health study psychopathology as a self-organized state emerging out of interdependent cognitive, affective, behavioral, and physiological processes [1–3]. This line of research has unarguably produced innovative and valuable insights into psychopathology, yet its most basic testable prediction has almost never been investigated: Do the observables of this complex system, i.e., psychological self-ratings, actually display *complex* dynamics? This is an important question to resolve, as it directly affects the veracity of the claim that psychopathology should be regarded as a complex system state. In the present study, we address this question by identifying characteristic markers of complexity in the dynamics of psychological observables as measured by repeated self-ratings of social, emotional, physiological, psychological, and behavioral states.

Recently, the mental health field is changing focus from group-based “disease models” to more personalized models of psychopathology. Group-based disease models view mental disorders as conditions in which a specific (biological or psychological) root cause leads to a set of observable symptoms [3]. In this view, knowledge about a population (e.g., people diagnosed with depression) can (at least to some extent) be generalized to an individual from that population. This assumption, however, does not hold when a population is heterogeneous (e.g., [4, 5]) or when processes are non-stationary (for example, when an individual’s mood is not stable over time) as is often the case in human sciences [6–9]. Because of the heterogeneity between patients and the intrinsic non-stationarity of clinical change processes, evidence-based practice guidelines based on group-level comparisons are only of limited use for the care of individual patients [10]. This is why scholars have called for a *personalized approach to psychopathology*, in which treatment is tailored to the specifics of the individual patient [11–14].

Recent advancements on the personalized approach to psychopathology pledge for a complex systems perspective on mental health [14–17]. This perspective has adopted metaphors and concepts from complexity science, a transdisciplinary research area originated in physics, mathematics, and biology [18–20]. The methods and analytic techniques of complexity science, however, are less commonly used in psychopathology research, although they have been used to study human behavior in the past [21–25]. Recent reviews of idiographic models and methods for psychopathology research did not include any of the available complexity methods [14, 26]. There thus appears to be a discrepancy between the assumed nature of the object under study (i.e., psychopathology as a state in a complex dynamical system) and the way it is scientifically studied.

In a complex dynamical system, the interdependencies between parts lead to the emergence of robust ordered states, or, patterns, at the level of the whole (see also [27, 28]). These patterns may function as *attractors* to the system, meaning that the system tends to maintain its current pattern despite perturbations from the internal or external environment (i.e., the system is attracted to a specific state). Several authors have proposed that psychopathological states function as attractors in a complex system comprising an individual in its environment [1, 2]. Such psychopathological attractors may be very entrenched, meaning that a patient is “stuck” in a particular state. The stability of attractors, however, can change over time, in which case alternative attractors become available to the system (i.e., multistability). Clinical improvement is then conceptualized as a so-called *phase transition* from a psychopathological towards a more healthy attractor state, while the onset of psychopathology is understood as the reverse. Recurrent depressive episodes may then for example be understood as phase transitions between a depressed and a healthy attractor.

Attractor states emerge through a process of self-organization: the direct interactions between the system parts with the internal or external environment (i.e., states emerge without a blueprint or latent cause [29, 30]). From a complex systems perspective, psychopathology is thus not a fixed condition with a root cause. Instead, it is a “soft-assembled,” self-organized, attractor state that arises out of—and is maintained by—the interactions between various component processes (e.g., biological, psychological, socio-cultural processes) whose dynamics evolve on different temporal and spatial scales. The complex systems approach to psychopathology provides a set of descriptive principles (e.g., attractors, feedback loops, self-organization) that are assumed to apply to all psychopathological states, while acknowledging that the content of these states (e.g., in terms of symptom profiles [4]) is highly individual and context specific as it has emerged from a unique history of interaction events. A complex systems approach thus naturally combines nomothetic and idiographic aspects of psychopathology [2].

Probably due to their intuitive appeal, complex systems concepts, such as attractors, have since long been around in clinical psychology [31–33]. These concepts, however, have mostly been used metaphorically and not literally in the form of complexity analysis of time series data (but see for early applications [34–36]). This is probably due to the fact that complexity science’s analytic toolbox has been relatively unknown to clinical researchers. In addition, these analyses require data not commonly collected in clinical research: time series data. A time series is a record of intensive longitudinal measurements of a variable, which describes the temporal evolution of an observable of a specific system. Typically, complexity analyses require time series with many measurement points, which are easily collected for physiological signals (e.g., heart rate), but traditionally not for psychological signals (e.g., the evolution of mood over time [37]). The rise of smartphones now make it possible to collect multivariate time series of such psychological signals, using methods like ecological momentary assessment (EMA) or experience sampling [38]. Also, advances on complexity analysis have led to methods that can be applied to relatively short time series as well (e.g., [39]). Thus, the complex systems approach to psychopathology is at a cusp to move beyond metaphors and to build a genuine complexity science of personalized psychopathology. The first step in this direction is to test whether time series of psychological self-ratings indeed exhibit characteristics of the dynamics of complex systems.

To answer this question, the present study will assess the presence of three characteristics of complex systems and their corresponding markers in time series data of psychological variables (see Table 1). First, complex systems develop over time and thus have *memory*. This means that the current state of a system depends on previous states. Rather than producing independent random fluctuations, observed values generated by a complex system are interdependent. Characteristic for complex self-organizing systems (such as humans) is that their memory is not limited to a short time scale (e.g., lag 1 or lag 2), but can in principle span any lag of time. Thus, long-term memory, identifiable as long-range temporal correlations and power-law scaling, is expected in the time series of a complex system as a sign of self-organized interactions across scales as has been evidenced in time series of human physiology and performance [40–43]. In a pioneering study, Delignières, Fortes, and Ninot [44] also found power-law scaling in bi-daily self-ratings of self-esteem and physical self-image. Moreover, temporal correlations are expected to be non-stationary (i.e., time-varying) in complex systems, which is considered the strongest evidence for multiplicative interactions across different temporal scales [45].

Second, complex systems exhibit phase transitions between attractor states: qualitative changes in the state of the system that are reflected in time series as *regime shifts* and non-stationarity [23, 46]. Different regimes refer to different attractors (e.g., a state of psychopathology and a state of health) and may be characterized by different mean levels (e.g., of symptom severity), different variance levels (e.g., emotional inertia vs. mood swings), or differences in any other distributional characteristic. Complexity theory predicts that phase transitions from one attractor to another are preceded by a period of instability, which comes apparent as a temporary increase of variability and complexity in time series data, thereby providing another source of non-stationarity [39, 47]. The study of these regime shifts, or broader defined, phase transitions, is an important avenue for clinical science. Scholars have argued that many important clinical events such as the onset of psychopathology [48], relapse [49], suicide attempts [50], clinical improvement [51–57] and clinical deterioration [51, 52] may be instances of phase transitions. Also, the instability that precedes such transitions can be detected as so-called early-warning signals (EWS) that may be used for short-term prediction of clinical change [55, 58].

Third, complex systems can show a *sensitive dependence on initial conditions*, which leads to a limited predictive horizon [59, 60]. This means that it is possible to predict time series of a complex system a few time points ahead, but not in the far future. The predictive horizon of a time series can therefore be useful to distinguish complex systems (strong prediction decay) from random systems (which are never predictable) and simple deterministic systems (which are always predictable). A well-known example of a complex system with a limited predictive horizon is the weather. Short-term prediction is possible, but prediction becomes highly unreliable after a few days. A clinical example is provided by Tschacher et al. [35], who found that the evolution of schizophrenia symptoms for many patients in their study sample was predictable only in the short term, but not in the long term (see also [34, 61, 62]).

The present study examines whether self-ratings collected with EMA exhibit the three signs of complexity introduced above: (1) time-varying short- and long-term memory, (2) regime shifts, and (3) sensitive dependence on initial conditions. We analyzed the presence of these complexity markers in a single-case dataset with long EMA time series (1476 time points) as a proof-of-principle.

**Table 1:** Characteristics of complex systems with corresponding markers and test procedures

Characteristic of complex system	Marker	Test
Memory	• Dependency on past values • Long-range temporal correlations • Non-stationary temporal correlations	• Bartels rank test • Inspect (partial) autocorrelation functions • Time-varying autoregressive model
Regime shifts	• Non-stationarity	• KPSS test for level stationary time series • Nonparametric change point analysis
Sensitive dependence on initial conditions	• Limited predictive horizon	• Forecast skill (Sugihara-May algorithm)

## Methods

### Dataset

Publicly available EMA data from the study “Critical Slowing Down as a Personalized Early Warning Signal for Depression” were analyzed [63, 64]. The dataset contains 1476 self-ratings over 239 consecutive days from a single individual. Data were collected during a double-blind experiment in which the participant reduced his dosage of anti-depressant medication. Twenty-nine items on momentary states of affect, symptoms, self-esteem, and physical sensations were rated on Likert and bipolar scales (ranging from 1 to 7 or from − 3 to + 3; items in Table 2). This was done multiple times per day during a baseline period, a period of dosage reduction, and after the dosage reduction. On average, the participant completed 6.2 (SD = 1.9) assessments per day. In addition, there were daily measures in the morning and evening regarding sleep and evaluation of the day, respectively. For a complete description of the dataset, see [63]. In the present study, we analyzed the twenty-nine items on momentary states. The time series of these items have 1476 observations per item. In total, 103 observations were missing, which is less than 1% of all observations (0.24%). The present dataset was chosen because of the long time series, which are necessary for several analysis techniques employed.

### Data analysis

The time series were analyzed for markers of complexity using the methods and tests listed in Table 1. This was done twice: once for the full measurement period and once for the “baseline period” only, consisting of 292 assessments that were completed when the participant was not yet tapering medication. We analyzed the baseline period separately in order to examine to what extent the complexity markers were present when clinical change (relapse into depression) was presumably absent. Data analysis of both measurement periods was done with missing data excluded and with missing data imputed by a multivariate imputation algorithm using chained equations [65]. Results were highly similar, likely because of the low number of missing values. We report here on the analysis with missing observations excluded. Each marker was calculated for each time series separately. When a statistical test was used, we corrected for multiple testing with Bonferroni correction by dividing the conventional alpha level of .05 by the amount of items tested and rounding to 3 decimals. A result was therefore considered significant when *p* < .002. All analyses were performed in R [66]. The materials to reproduce the present analysis are available at the open science framework (https://osf.io/nca2u/). We elaborate on the specific tests in the “Results” section.

**Table 2:** Descriptive statistics and results of the analysis

	Descriptive statistics		Bartels rank test H0 = random H1 = non-random		Significant partial autocorrelation		EDF of TV-AR		KPSS test H0 = level stationary H1 = unit root		Number of significant change points	
Item	Mean	SD	All data	Subset	*N*	Max lag	All data	Subset	All data	Subset	All data	Subset
I feel relaxed	4.17	0.75	< .001*	< .001*	17	932	3.36*	8.35*	.097	.029	5	2
I feel down	3.18	0.74	< .001*	< .001*	27	402	2.00*	2.00*	.010*	.100	5	0
I feel irritated	2.24	1.17	< .001*	< .001*	19	667	3.28*	2.00*	.010*	.050	1	0
I feel satisfied	4.2	0.99	< .001*	< .001*	17	478	9.40*	3.06*	.100	.012	2	0
I feel lonely	3.01	0.49	< .001*	< .001*	19	606	9.14*	2.00	.010*	.089	1	0
I feel anxious	3.09	0.31	< .001*	< .001*	29	594	8.95*	4.53*	.010*	.100	2	0
I feel enthusiastic	3.83	0.86	< .001*	< .001*	16	379	2.00*	2.50*	.100	.090	0	0
I feel suspicious	1.26	0.55	< .001*	< .001*	31	405	9.17*	2.94	.010*	.052	7	0
I feel cheerful	4.09	0.84	< .001*	< .001*	24	406	2.00*	3.05*	.100	.043	6	0
I feel guilty	3.01	0.46	< .001*	< .001*	19	886	9.50*	2.58	.010*	.010*	6	1
I feel indecisive	1.85	0.87	< .001*	< .001*	25	750	2.79*	2.00*	.100	.010*	11	1
I feel strong	4	0.87	< .001*	< .001*	24	826	4.38*	2.00*	.100	.019	8	0
I feel restless	2.04	0.93	< .001*	< .001*	24	617	9.72*	5.75*	.010*	.050	5	0
I feel agitated	2.13	0.98	< .001*	< .001*	20	819	9.62*	2.00*	.010*	.045	5	0
I worry	1.34	0.84	< .001*	< .001*	30	547	2.00*	2.00*	.010*	.089	2	0
I can concentrate well	4.37	0.72	< .001*	< .001*	17	431	2.00*	2.66*	.010*	.010*	1	1
I like myself	4.66	0.57	< .001*	< .001*	18	552	7.23*	2.51*	.100	.010*	6	1
I am ashamed of myself	1.22	0.59	< .001*	< .001*	19	716	2.00*	2.33	.010*	.100	1	0
I doubt myself	1.99	0.92	< .001*	< .001*	21	733	3.00*	2.00*	.058	.100	8	0
I can handle anything	3.94	0.79	< .001*	< .001*	19	466	2.00*	2.33	.064	.043	1	0
I am hungry	1.46	0.73	.068	.068	13	484	3.40*	4.37	.010*	.014	0	0
I am tired	2.01	0.82	< .001*	< .001*	23	668	9.75*	2.91	.010*	.100	5	0
I am in pain	1.34	0.53	< .001*	< .001*	14	571	9.14*	3.04	.100	.027	2	0
I feel dizzy	1.01	0.08	.854		24	858	3.03		.010*		0	
I have a dry mouth	1	0.04	.958		18	702	3.59		.029		0	
I feel nauseous	1.01	0.08	.854		21	942	2.00		.100		0	
I have a headache	1.43	0.65	< .001*	.854	19	710	8.49*	8.35	.022	.010*	9	1
I am sleepy	1.45	1.01	< .001*	.958	16	241	6.99*	2.00	.010*	.016	1	0
From the last beep onwards I was physically active	1.94	0.93	< .001*	.854	16	685	3.37*	2.00	.010*	.100	1	0
Sum of significant tests (%)			25 (86%)	22 (85%)			26 (89%)	15 (58%)	16 (55%)	4 (19%)		

*N* number. *N* = 1476 for all data. *N* = 292 for the subset. *Statistically significant test statistics (*p* < .002). Descriptive statistics were calculated with all ratings scaled to a range from 1 to 7. For Bartels rank test, results were considered significant for *p* < .002. The KPSS test only provides *p* values between .010 and .100. For the KPSS test, *p* = .010 was therefore considered significant. Three items showed no variance during the baseline period included in the subset and were therefore omitted from analysis of the subset

## Results

### Memory

#### Dependency

Memory in its most general form is reflected in time series as dependence on past values. Statistically, this means that time series generated by a complex system are non-random. We tested for randomness using Bartels rank test. In both the full measurement period and the baseline period, ~ 85% of the items were shown to be non-random (Table 2). The results showed that all measures of psychological variables were non-random. In contrast, for several measures of physical sensations, such as the item “I am hungry,” the null hypothesis of randomness was not rejected.

#### Long-range temporal correlations

**Fig. 1:**
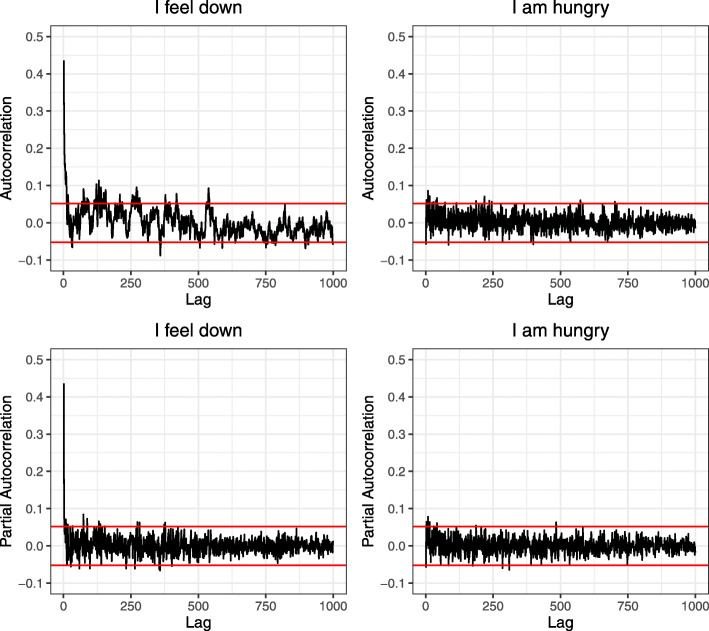
Autocorrelation functions and partial autocorrelation functions of the items “I feel down” and “I am hungry.” The red line indicates significance threshold with *p* < .05

We studied long-term memory by inspecting long-range temporal correlations. We plotted the autocorrelation function (ACF) for each item. The ACF shows the correlation of a time series with a lagged version of itself. The ACF can be visualized by plotting the correlation strength against the lag size (see Fig. 1 for an example; ACF plots for all items are available online at the open science framework). Visual inspection of the ACFs shows that both short-term (e.g., lag 1, lag 2) and long-term memory are common in the present time series. There are, however, considerable differences in ACFs between items. For example, in Fig. 1, the ACF for a typical psychological variable (“I feel down”) can be compared to the ACF for “I am hungry.” While “I feel down” shows both short-term and long-term memory, “I am hungry” shows little short-term memory and no clear pattern of long-term correlations. To summarize the presence of long-term memory, we calculated the number of lags with significant partial autocorrelations per item (i.e., correlation estimates at higher lags are corrected for autocorrelation at lower lags). The partial autocorrelation function was calculated with the function pacf() as implemented in the stats package of R [66]. The significance threshold was based on a two-tailed *Z* test with the time series length as number of observations. Results showed that all items had significant partial autocorrelations (Table 2). For each item, significant partial autocorrelations were also found at very high lags (i.e., > lag 200; Table 2).

#### Non-stationary temporal correlations

**Fig. 2:**
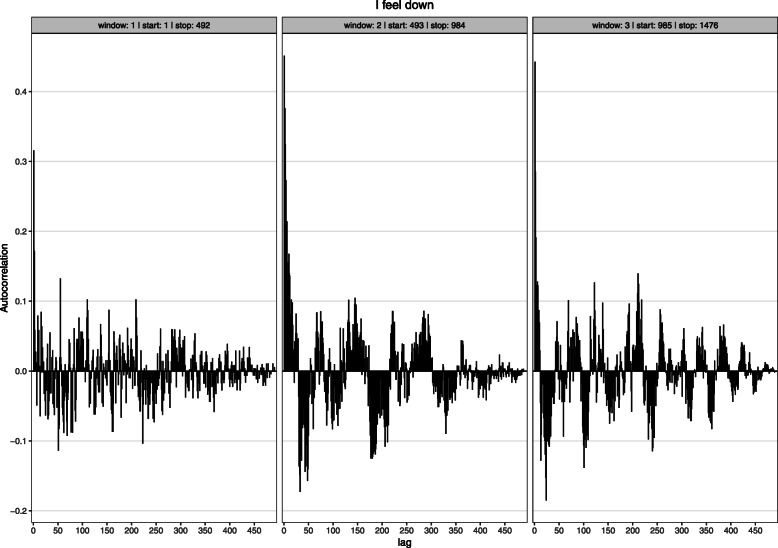
Three autocorrelation functions of the item “I feel down” calculated in a non-overlapping moving window of size 492

**Fig. 3:**
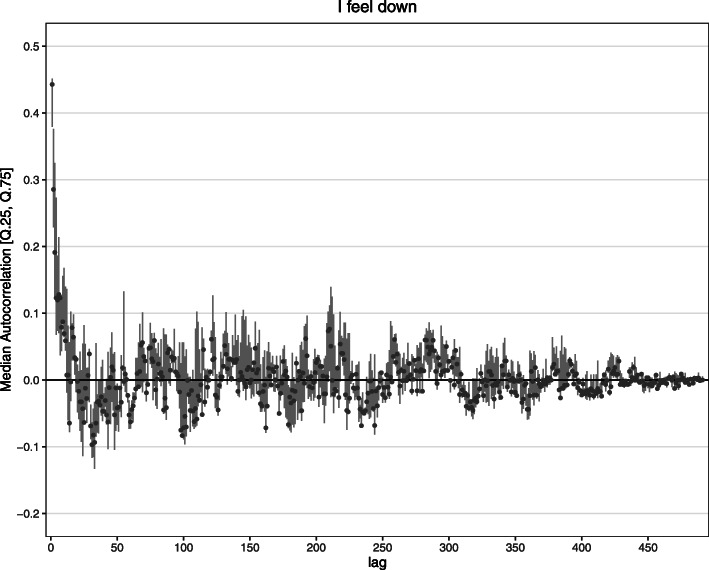
Median autocorrelation (points) with range between 25th and 75th quantile (lines) by lag for the item “I feel down.” Autocorrelation functions were computed in 985 overlapping moving windows of size 492

We tested non-stationarity in the lag 1 autocorrelation with a time-varying autoregressive (TV-AR) model, using the R-package MGCV [67]. The TV-AR model uses nonparametric smooth functions to model time-varying autoregressive coefficients at specific lags. We examined a TV-AR model for lag 1, because it is the most examined lag in EMA research and often shows the strongest autocorrelation (e.g., [68]). Non-stationarity of the lag 1 autocorrelation was evaluated by the significance test of the TV-AR (which tests whether the smoothing time-varying function is different from zero) and the effective degrees of freedom (EDF) of the smoothing function. The EDF indicates the number of parameters needed to represent the smoothing function [69]. An EDF of 2 indicates a linear trend of autocorrelation over time, which can either mean that the autocorrelation is not changing or changing linearly. An EDF higher than 2 indicates that the autocorrelation is definitely non-stationary. The results show that the lag 1 autocorrelation is often non-stationary (Table 2). Visual inspection of the fit of the TV-AR model shows that items with an EDF of 2 often had a non-stationary (increasing or decreasing) lag 1 autocorrelation as well (see supplemental figures TV-AR available at the open science framework). The autocorrelation function can also be non-stationary at different lags than 1. We explored this visually by plotting the autocorrelation function in a moving window of 492 time points. These plots show that the autocorrelation function is often non-stationary at many different lags (see Fig. 2 for an example; moving window ACF plots for all items are available online at the open science framework). We also visualized the variation of autocorrelation coefficients per lag (see Fig. 3 for an example; visualizations for all items are available online at the open science framework).

### Regime shifts

Phase transitions can be reflected in time series data as regime shifts: distributional changes in the time series including changes in mean and variance. Statistically, this leads to non-stationarity. We tested the hypothesis that time series could be seen as stationary around a level using the KPSS test as available in the R-package tseries [70]. The alternative hypothesis of the test is the presence of a unit root: a systematic unpredictable pattern, indicating non-stationarity. Results are presented in Table 2. Non-stationarity was found in many items when tested over the whole measurement period and in few items for the baseline period. Additionally, we estimated the number of regime shifts with a change point analysis, specifically the e.divisive algorithm, available in the R-package epc [71], which identifies significant changes in the distribution of data points over time. E.divisive compares all possible data segments and tests significance differences using a permutation test. The results show that many items have multiple change points, especially when the full measurement period is considered. In the baseline period, one can see largely converging results from the non-stationarity test and the change point analysis.

**Fig. 4:**
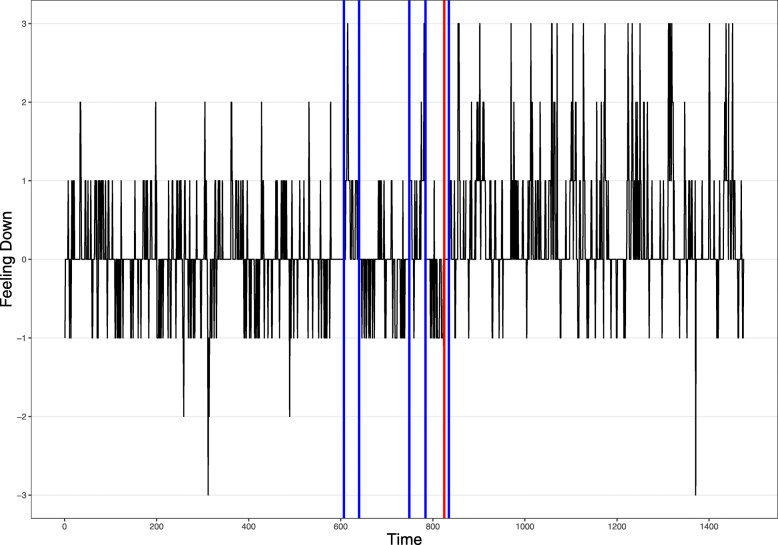
Change point analysis for the item “I feel down.” Blue vertical lines indicate change points in the time series. The red vertical line indicates measurement point 825, corresponding to day 127 around which the transition towards depression was identified in the weekly symptom measures [64]

Visual inspection of the results from the change point analysis suggests a relation between regime shifts in the time series of some items and the transition towards a depressive episode that the participant experienced. This is clearly illustrated for the item “I feel down” (Fig. 4) that was also found to be non-stationary (Table 1). One can see that the transition towards depression (Fig. 4; red line) is preceded by a period of instability in the time series, in which the item “I feel down” flickers between two regimes, one associated with lower scores and one with higher scores. After the transition towards the depressive episode, the time series settles in the regime with higher scores. Notably, the time series of the other items sometimes show regime shifts that seem related to the transition towards depression (as in Fig. 4), but there are also many regime shifts during presumably more stable periods in the participant’s mental state (figures of change points for all items are available online at the open science framework).

### Sensitive dependence on initial conditions

Sensitive dependence on initial conditions means that an extremely small difference between two trajectories at present can lead to dramatically large differences in the future, i.e., a divergence of trajectories. This divergence is the result of nonlinearity, a property that is typical for complex systems. As a consequence of sensitive dependence on initial conditions, complex systems tend to produce trajectories that are predictable only on the short term. Predictability can therefore be used to distinguish between different types of systems. Random systems have no predictability at all, simple deterministic systems (e.g., a sine wave) are perfectly predictable both short and long term. Complex systems lie in between these extremes: the predictability of their trajectory quickly decays over time.

#### Phase space reconstruction

As a measure of predictability, we computed forecast skill using the procedure proposed by Sugihara and May [72] using the R-package rEDM [73]. First, time series are embedded in a reconstructed phase space in which time-lagged copies of the time series are used as dimensions. The method of phase space reconstruction is based on Taken’s theorem [74]. This theorem entails that, under a limited set of assumptions, the state space dynamics of the system as a whole can be retrieved (under topological equivalence) from the observation of a single dimension of its state space: As long as the dimensions of the state space of a multidimensional dynamic system can be represented by coupled interdependent processes, its behavior can be reconstructed from a time series of only one observable dimension of that system. The state evolution of the system can be thought of as a trajectory through its state space. This trajectory is predicted by the Sugihara-May algorithm. The algorithm also imputed the few missing observations, as part of this specific analysis.

#### Procedure

The data were split in two parts. From the first 700 data points, the trajectory of each item in its reconstructed phase space was used to predict the trajectory of each item in a reconstructed phase space of the remaining measurement period. To create time-lagged copies of the time series as surrogate dimensions, we used a time lag of 4, which was the median optimal time lag for all items. The determination of the time lag is an optimization procedure, but in principle, every time lag will do. We used 13 dimensions in the embedding procedure, as this was the maximum optimal embedding dimension for all items. Too few dimensions is problematic, because it can lead to false identifications of similar trajectories [75]. It is not problematic to have too many dimensions. A measure of predictability, *forecast skill*, is then computed as the Pearson correlation between values that are predicted by the Sugihara-May algorithm and the observed values.

#### Forecast skill

**Fig. 5:**
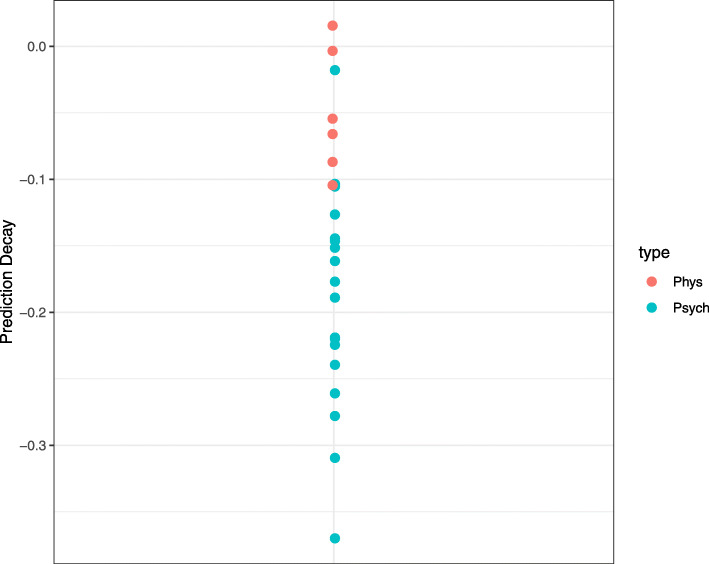
Slopes of forecast skill over time. Values indicate how strong the forecast skill decreases in 1 time step, calculated over the first five time steps

**Fig. 6:**
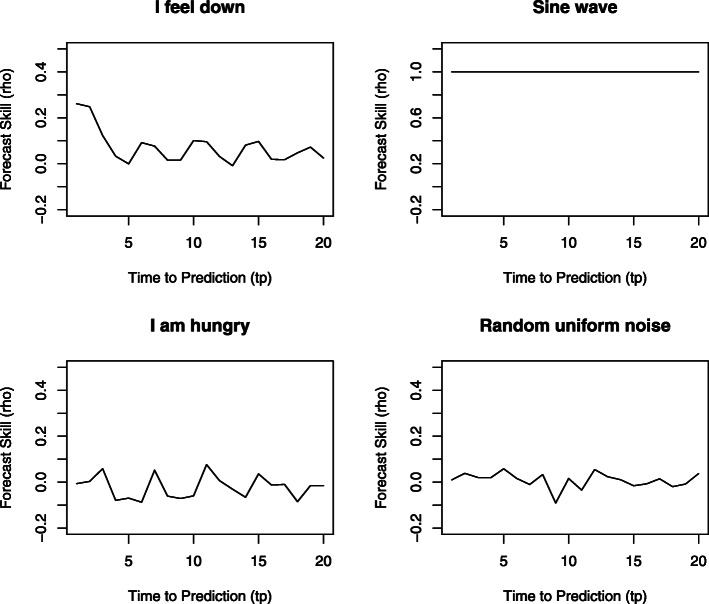
Forecast skill over time plotted for the items “I feel down” (upper left), “I am hungry” (lower left), a sine wave (upper right), and random uniform noise (lower right). The item “I feel down” shows a limited predictive horizon with a strong prediction decay that is characteristic for complex systems. The contrast is clearly seen with a completely predictable system (the sine wave) and a completely random system (the random uniform noise). The item “I am hungry” shows a prediction decay that more closely resembles a random system than a complex system

Forecast skill was compared at different time lags. A decrease in predictability over time lags is called a *prediction decay*. As a measure of prediction decay, we computed the slope of forecast skill over time within the first 5 lags (which was for most items a linear area in the forecast skill plots; see all forecast skill plots online at the open science framework). Our results show that for almost all items, forecast skill decreased over time (Fig. 5). However, there were interesting differences between items. Psychological items tended to show a clear decrease in predictability over time, suggesting that these time series indeed present the dynamics of a complex system. Items related to physical sensations, such as the item “I am hungry,” however, tended to show very low predictability both short and long term, suggesting that these time series are random. In Fig. 6, forecast skill is plotted against time for a typical affect item, a typical physical item, a sine wave, and random uniform noise. One can see that the predictability of “I feel down” decreases rapidly over time, as is expected for time series generated by complex systems. In contrast, “I am hungry” is never predictable and behaves as a random variable.

## Discussion

The present study examined whether the dynamics of repeated self-ratings are *complex* dynamics. We analyzed long psychological time series collected with EMA for three markers of complexity: (time-varying) short- and long-term memory, regime shifts, and sensitive dependence on initial conditions. Results showed that the present self-ratings were indeed complex: almost all items exhibited clear evidence for the three markers.

First, we found that the analyzed time series possessed memory: current values depended on past values. This was not restricted to short-term memory in the form of a dependency on a previous assessment from hours or a day ago (e.g., lag 1, lag 2). Current values also correlated with values in the distant past that were assessed weeks or months ago (e.g., lag 200, lag 1000). These long-range temporal correlations are indicative of interactions across scales and self-organization in complex systems [43, 45]. Long-range temporal correlations can be understood as slow “waves” that are reflected by the time series [45, 76]. The present results align with the ever growing empirical record of studies that have evidenced the presence and characteristics of long-range correlations and fractal scaling (i.e., a specific pattern of long-range correlations associated with self-organization [42]) in time and trial series of human physiology and performance [40–44, 77–86]. Moreover, the temporal correlations changed over time, indicating multifractality, which is considered a strong indicator for multiplicative interactions across time scales as data-generating process [45]. In sum, these results go against the notion that repeated self-ratings are a collection of independent, memoryless [87], observations. Instead, repeated self-ratings exhibit complex temporal patterns yielding time-varying short- and long-term memory.

Second, about half of the time series were non-stationary and many time series exhibited multiple change points, indicating the presence of regime shifts. This reflects the occurrence of phase transitions: qualitative shifts from one attractor state to another. In the current dataset, the relapse of the participant towards a depressive state was an example of such a phase transition, which was related to a regime shift in weekly symptom scores in a previous study [64]. This phase transition has also led to non-stationarity and change points in EMA items (e.g., Fig. 4). Change point analysis showed, however, that many items had more than one change point, suggesting that multiple phase transitions occurred in the specific psychological processes that were measured with EMA (e.g., the flickering observed in Fig. 4). When stationarity and change points were only examined during the baseline period of the experiment in which medication was not yet being tapered, only few items showed evidence for regime shifts.

Third, the time series showed a limited predictive horizon, signaling the sensitive dependence on initial conditions that is typical for complex systems. The predictive horizon seemed to be limited to about 3 to 5 time points. The observed prediction decay indicates the presence of a butterfly effect: the trajectories of once closely located data points completely diverge over time. Thus, while short-term prediction of these repeated self-ratings is possible, these results suggest that long-term prediction may be fundamentally impossible. In the current analysis, trajectories were compared within the time series of the same person, but the butterfly effect applies equally well to trajectories of different persons. In a recent study by Rubel et al. [88], process-outcome relations in psychotherapy could not be reliably predicted on the basis of process-outcome relations from patients with highly similar pretreatment characteristics. A finding which may be explained by the presence of a butterfly effect (i.e., individual trajectories diverge over time; see also [61, 62]).

One interesting and unexpected result was the difference between self-ratings of psychological states and physical sensations in the presence and strength of markers of complexity. All psychological states, such as “I feel down,” were found to be non-random in both the full dataset and the subset. In contrast, for physical sensations, such as “I am hungry,” the null hypothesis of randomness was often not rejected, at least not in the subset. Moreover, when looking at the predictive horizon in the forecast skill graphs, the psychological states all showed the typical prediction decay that characterizes complex systems, while the physical sensations showed a prediction horizon that more closely resembles that of a random variable. Physical sensations, such as hungriness, are of course not truly random processes. This result should thus be explained either by the appropriateness of the sampling rate in this study for the different variables or by the nature of the variables themselves (e.g., “I am hungry” may refer to an unambiguous identifiable internal state that can be thought of as being experienced at different orders of magnitude, “I feel suspicious,” much less so, see also [89]). Either way, the striking difference between the two types of items may be an important avenue for future research, with the potential to shed more light on the nature of psychological measurement.

In sum, the present study corroborates the assumption that self-ratings of psychological states display complex behavior and support the proposal that a complex systems approach to psychopathology is warranted [1–3, 90, 91]. The present study is limited to a single case with long time series, which enabled us to thoroughly examine the dynamics and assess multiple markers with multiple techniques. The single-case design, however, can by definition not be used to estimate the frequency of a phenomenon (in this case how many individuals will exhibit complex behavior in their psychological dynamics). Rather, the single-case study shows the existence of a phenomenon. Thus, the results primarily indicate that complexity in psychological dynamics should be considered. However, given the strong theoretical arguments for humans to be complex (and not random or simple deterministic) systems [28] and the omnipresence of complexity in other human-generated time series [43], we judge it unlikely that the complexity in the present dataset is a coincidence. Instead, the present results can be seen as illustrating the need for a complex systems approach to psychopathology that moves beyond metaphors and appreciates the scientific and clinical implications of complexity.

### Scientific implications

First, the presence of both short- and long-term memory has methodological implications. Widely applied statistical time series models of psychological dynamics, i.e., autoregressive (AR) models, do often not account for long-term memory [14, 26]. In these models, the current value of a variable is typically predicted by values in the near past (usually lag 1, or lag 2; e.g., [68]), but not in the distant past. Not taking into account long-range temporal correlations can “completely invalidate statistical inference” [92]. However, taking these long-range temporal correlations into account in a statistical model is far from straightforward. In the current study, we found for many items significant partial autocorrelations in as much as 20 different lags. In an AR model, these would be modeled as 20 independent, additive processes that influence the current state of the system. In other areas of psychological science, there is much debate about whether this is a sensible model to describe a time series [45, 93–95]. Proponents of AR models argue that the different autocorrelations represent distinct additive causal processes [95]. Complexity scientists argue that they do not, and propose the presence of long-range correlations to be a consequence of self-organized interactions between interdependent (i.e., non-additive) causal processes at different temporal scales [96]. The latter argument is supported by the fact that long-range correlations are ubiquitous in nature, even when distinct additive causal processes are clearly absent (e.g., self-organization in a pile of sand [41]). Moreover, data with non-stationary autocorrelation functions, as we observed, cannot be generated by the most flexible AR model (i.e., the autoregressive fractally integrated moving average model [97]), but can be generated by models that feature interaction across scales (i.e., cascade models [45]). Thus, following a complex systems perspective that embraces self-organization as the causal mechanism, statistical models of time series can only be used descriptively and not as formal models of the underlying causal processes (see also [98]).

Vector autoregressive (VAR) models are time series models that include multiple variables that may predict themselves and each other at specific lags (often restricted to lag 1 in psychopathology research). VAR models are used to examine Granger causality [99]. Granger causality entails that if a variable *X* at one time point predicts a variable *Y* at a future time point beyond the autoregressive effect of *Y* (how *Y* predicts itself), *X* can be said to be Granger causal for *Y*. Although researchers using VAR models are careful in interpreting their models in causal terms, the general idea of these models is that they at least can give a hint of possible causal relations. Accordingly, variables that are Granger causal for many other variables are suggested as targets for treatment (e.g., [68]). This reasoning, however, is problematic under conditions of complexity. Due to the presence of short- and long-term memory, time series are not stochastic beyond what is typically modeled by the VAR model, meaning that Granger causality does not apply [99]. Sugihara et al. [100] illustrate that the (lagged) correlations between variables in a complex (eco)system are indeed bad indicators for causality. Two variables that have a stable causal connection in a complex system may exhibit non-stationary correlation with each other, even to the point that the correlation coefficient switch signs [100]. In a simulation study on psychological dynamics, Haslbeck and Ryan [101] found that a VAR model cannot retrieve the complex systems model that generated the data. Sugihara et al. [100] propose convergent cross mapping as a possible alternative for Granger causality. Also, recurrence-based analysis, which requires no assumptions concerning the structure of the data, may be a promising approach to investigate the dynamics of EMA data [89, 102, 103]. Future research should further examine these techniques for psychological self-ratings.

Second, complex systems exhibit phase transitions, which lead to regime shifts and thus non-stationarity in time series data. While regime shifts are generally undesirable in statistical approaches to psychological dynamics [26], they are an important research avenue in a complex systems approach. In the dynamic research strategy outlined by Thelen and Ulrich [23], phase transitions form *the* starting point for the study of developmental and also clinical change.

- First, phase transitions differentiate between different attractor states that demand their own descriptions. For example, a depressed attractor state may yield a specific pattern of cognition, emotion, behavior, and physiology that is reflected in specific values of such variables (e.g., high negative emotions, high rumination) as well as the interrelations between them (e.g., a feedback loop between negative thoughts and negative feelings). A healthy attractor of the same person may in contrast be characterized by completely different values and interrelations of these variables [1]. Aggregation of a time series over different regimes should thus be avoided as this will give a misleading impression of the patient’s actual psychological states (e.g., emotions may appear neutral on average, while the patient actually experienced one period of negative emotions and one period of positive emotions). Future research should therefore explicitly examine possible different attractor states in time series (for example with change point analysis).
- A second research avenue is the study of early-warning signals (EWS) as precursors of upcoming phase transitions [104]. In a study on self-ratings collected during psychotherapy, EWS were indeed shown to be predictive for upcoming regime shifts in symptom severity levels [52]. Such real-time prediction of transitions may be highly relevant for prevention and intervention in clinical practice. During periods of EWS, complex systems are more sensitive to external influences. Interventions, aimed at either eliciting positive change (e.g., sudden gains [52];) or preventing negative change (e.g., suicide attempts [50];), may thus be increasingly effective during periods of destabilization (see also [105]). Future research should test this hypothesis.
- A third reason to study phase transitions is that these are the moments at which change mechanisms in complex systems may be revealed [23, 106]. Consider a simple example: the emergence of convection rolls when boiling water. At a critical temperature, where the body of water cannot dissipate its heat any more in a regular fashion, convection rolls emerge spontaneously. At the transition point, a change mechanism (a control parameter in complex systems terms) can be identified: the kinetic energy (i.e., heat) that is delivered to the pan. This sounds obvious but note that the heat may not have been identified as the control parameter if the water was only observed within a small temperature range that did not include the critical threshold, or the tipping point. Control parameters thus only become apparent as change mechanisms at the tipping points of transition. From this perspective, EMA research is thus especially interesting during a change process (e.g., therapy) and not during a baseline period in which the change mechanisms are likely to remain hidden. In psychopathology, control parameters will of course be harder to identify than in the example of convection rolls described above. Control parameters for clinical change are likely to be individual and contextualized. This makes control parameters an interesting avenue for applied idiographic research (for steps in this direction, see [107–109]).

Third, complex systems show sensitive dependence on initial conditions, which implies that psychological dynamics are predictable in the short term, but not in the long term. For clinical science, this means that long-term prediction of individual trajectories may be fundamentally impossible. Instead, researchers should aim at short-term prediction and be careful in forecasting further in the future as predictions will become increasingly unreliable. Short-term prediction is a promising future direction for intervention science, as it may enable just-in-time interventions (e.g., [110]). It should be noted, however, that advancements are made in deriving the governing equations of complex systems from time series data, in which case long-term prediction would in theory become possible (e.g., [111]). Future research could explore the possibility to derive such equations from EMA data.

### Clinical implications

If we accept the three markers and their meaning in complex systems theories as fundamental aspects of psychopathology and clinical change, this has several implications for clinical practice and public health. Note that, in contrast to the scientific implications given above, these implications are more on a conceptual level, rather than a data level, and therefore arguably more speculative. Our primary aim is to illustrate how the clinical implications of a complex systems approach, as we and others have proposed elsewhere [1, 2, 90, 91], relate to the three characteristics that we examined.

First, our findings regarding memory support the assumption that psychopathology emerges from self-organized interactions between processes at different temporal scales [90]. Current psychopathology is then the result of a unique life-span history of interaction events, which can explain why psychopathology is highly individualized [4, 14]. For clinical practice, this implies that classification is fundamentally limited for clinical case formulation (although it may fulfill a practical function). Instead, personalized case conceptualization should be preferred [12].

Second, our findings regarding regime shifts support the idea that clinical change represents a phase transition from one attractor to another (e.g., a transition into a depressive episode). Therapeutic change is then expected to be discontinuous and irregular rather than continuous and gradual. Also, this means that dose-response relations in psychopathology will often be disproportional [112]: when a patient is in an entrenched psychopathological attractor, interventions will have very little effect, but if a patient is close to a tipping point, small interventions can have enormous effects. The hypothesis that treatment is increasingly effective during destabilization periods is an important avenue for future research. The generic principles of therapeutic change by Schiepek et al. [2] and the network destabilization and transition model by Hayes et al. [1] provide two (related) process-oriented frameworks for how clinicians may support phase transitions towards clinical improvement in psychotherapy. Central to these approaches is that treatment does not follow a strict protocol, but is personalized to the dynamic state of the patient (e.g., in a stable attractor or a destabilization period).

Third, as exemplified by sensitive dependence on initial conditions, we expect clinical change processes to follow complex individual pathways that are very hard to predict based on baseline characteristics, rather than standard tracks [61]. On the individual level, clinical change processes are highly irregular and fluctuating, as comes apparent when enough measurements are taken. Too few measurements (e.g., only pre and post) can then be misleading, and frequent process monitoring (e.g., with daily self-ratings) is essential for a valid measure of the change processes [113] (for an overview of all scientific and clinical implications, see Table 3).

**Table 3:** Characteristics of complex systems with corresponding scientific and clinical implications

Characteristic complex system	Scientific implications	Clinical implications
Memory	• Absence of long-range temporal correlations and stationarity of temporal correlations cannot be assumed, but should be examined • The data-generating process of EMA data is likely to involve interactions across scales • Future research should explore techniques that do not make assumptions about the correlation structure of EMA data such as recurrence analysis or convergent cross mapping	• Current psychopathology should be understood as emergent from a life-span history of interaction events • Patients’ specific psychopathological states are fundamentally individualized
Regime shifts	• Stationarity of mean and variance cannot be assumed, but should be examined • Different regimes in a time series demand their own description • Future research should further study drivers and predictors of phase transitions	• Enduring clinical improvement may be understood as a phase transition • Successful treatments are then characterized by a destabilization period in which the patient’s psychological state is more variable • Interventions are hypothesized to be more effective during periods of destabilization
Sensitive dependence on initial conditions	• Long-term prediction of psychological self-ratings may be fundamentally impossible • Future research should focus on short-term prediction	• Frequent process monitoring is essential to track the change process • Few measurements may give a misleading impression of the clinical change processes

### Outlook

Last, we propose two (complementary) directions for future research: a nomothetic and an idiographic approach. First, the nomothetic approach to psychopathology research should aim at identifying and understanding general properties of clinical change based on the principles of complex systems (e.g., phase transitions). These general principles govern laws of within-system changes and thus cannot be derived from traditional group-level research but by drawing generalizations across cases, possibly in a multi-level framework ([9]; e.g., [52]). Research questions in this approach are as follows: how is destabilization related to clinical change? Can we predict phase transitions? Are interventions more effective during periods of destabilization?

Second, we propose an idiographic approach which, informed by nomothetic research, should aim at studying the manifestation of these general principles in single individuals, thereby providing individualized and contextualized content. Research questions in this approach are as follows: what does the psychopathological state of a patient entail? What feedback loops might play a role in maintaining this state? What might be possible control parameters that drive a client to its tipping point towards a more healthy psychological state? Such an idiographic approach, informed by general principles, provides an excellent starting point for applied clinical research which may directly inform clinical decision-making (for steps in this direction, see, e.g., [108, 110, 113]).

## Conclusions

The present study illustrates that complexity should be considered in the study of psychological self-ratings. This finding highlights the need to adopt principles of complex systems theory and methods into psychopathology research. As an outlook, we envision a complexity science of psychopathology that bridges the gap between nomothetic and idiographic research and between science and practice.

## Data Availability

The dataset analyzed during the current study is available at the open science framework repository [https://osf.io/j4fg8]. The open materials are available at the open science framework repository [10.17605/OSF.IO/NCA2U; https://osf.io/nca2u/].

## Abbreviations

ACF: Autocorrelation function
AR: Autoregressive
EMA: Ecological momentary assessment
EWS: Early-warning signals
TV-AR: Time-varying autoregressive
VAR: Vector autoregressive

## References

[1] Hayes AM, Yasinski C, Ben Barnes J, Bockting CLH (2015). Network destabilization and transition in depression: new methods for studying the dynamics of therapeutic change. Clin Psychol Rev.

[2] Schiepek G, Eckert H, Aas B, Wallot S, Wallot A (2016). Integrative psychotherapy: a feedback-driven dynamic systems approach.

[3] Borsboom D, Cramer AOJ (2013). Network analysis: an integrative approach to the structure of psychopathology. Annu Rev Clin Psychol.

[4] Fried EI, Nesse RM (2015). Depression is not a consistent syndrome: an investigation of unique symptom patterns in the STAR∗D study. J Affect Disord.

[5] Wolfers T, Doan NT, Kaufmann T, Alnæs D, Moberget T, Agartz I, et al. Mapping the heterogeneous phenotype of schizophrenia and bipolar disorder using normative models. JAMA Psychiatry. 2018:1–10. 10.1001/jamapsychiatry.2018.2467 PMC6248110 30304337

[6] Rose T (2016). The end of average: how to succeed in a world that values sameness.

[7] Fisher AJ, Medaglia JD, Jeronimus BF. Lack of group-to-individual generalizability is a threat to human subjects research. Proc Natl Acad Sci. 2018;115. 10.1073/pnas.1711978115 PMC6142277 29915059

[8] Molenaar PCM (2009). A manifesto on psychology as idiographic science: bringing the person back into scientific psychology. Time Forever Measurement.

[9] Hamaker EL, Mehl MR, Conner TS (2012). Why researchers should think “within-person”: a paradigmatic rationale. Handbook of research methods for studying daily life.

[10] Van Os J, Guloksuz S, Vijn TW, Hafkenscheid A, Delespaul P (2019). The evidence-based group-level symptom-reduction model as the organizing principle for mental health care: time for change?. World Psychiatry.

[11] Hofmann SG, Hayes SC (2019). The future of intervention science: process-based therapy. Clin Psychol Sci..

[12] van Os J, Delespaul P, Wigman J, Myin-Germeys I, Wichers M (2013). Beyond DSM and ICD: introducing precision diagnosis for psychiatry using momentary assessment technology. World Psychiatry.

[13] Fisher AJ (2015). Toward a dynamic model of psychological assessment: implications for personalized care. J Consult Clin Psychol.

[14] Wright AGC, Woods WC. Personalized models of psychopathology. Annu Rev Clin Psychol. 2020;16. 10.1146/annurev-clinpsy-102419-125032 32070120

[15] Schiepek G (2003). A dynamic systems approach to clinical case formulation. Eur J Psychol Assess.

[16] Fisher AJ, Newman MG, Molenaar PCM (2011). A quantitative method for the analysis of nomothetic relationships between idiographic structures: dynamic patterns create attractor states for sustained posttreatment change. J Consult Clin Psychol.

[17] Borsboom D (2016). A network theory of mental disorders. World Psychiatry.

[18] Haken H (1983). Synergetics: an introduction. Non-equilibrium phase transition and self-selforganisation in physics, chemistry and biology.

[19] Prigogine I, Stengers I (1984). Order out of chaos: man’s new dialogue with nature.

[20] Gilmore R. Catastrophe theory. Applied Physics 1992. p. 85–119.

[21] Kelso JAS, Schöner G, Scholz JP, Haken H (1986). Phase-locked modes, phase transitions and component oscillators in biological motion. Phys Scr.

[22] Stephen DG, Dixon JA, Isenhower RW (2009). Dynamics of representational change: entropy, action, and cognition. J Exp Psychol Hum Percept Perform.

[23] Thelen E, Ulrich BD (1991). Hidden skills: a dynamic systems analysis of treadmill stepping during the first year. Monogr Soc Res Child Dev.

[24] Van der Maas HL, Molenaar PC (1992). Stagewise cognitive development: an application of catastrophe theory. Psychol Rev.

[25] Guastello SJ, Koopmans M, Pincus D (2008). Chaos and complexity in psychology: the theory of nonlinear dynamical systems.

[26] Piccirillo ML, Rodebaugh TL (2019). Foundations of idiographic methods in psychology and applications for. Clin Psychol Rev.

[27] van Geert PLC (2019). Dynamic systems, process and development.

[28] Ladyman J, Lambert J, Wiesner K (2013). What is a complex system? Vol. 3. Eur J Philos Sci.

[29] Haken H. Synergetics in psychology. In: Tschacher W, Schiepek G, Brunner EJ, editors. Self-organization and clinical psychology Springer Series in Synergetics, vol 58. Berlin: Springer; 1992. p. 32–54.

[30] Haken H (2006). Synergetics of brain function. Int J Psychophysiol.

[31] Hayes AM, Strauss JL (1998). Dynamic systems theory as a paradigm for the study of change in psychotherapy: an application to cognitive therapy for depression. J Consult Clin Psychol.

[32] Schiepek G, Tschacher W. Application of synergetics to clinical psychology. In: Tschacher W, Schiepek G, Brunner EJ, editors. Self-organization and clinical psychology Springer Series in Synergetics, vol 58. Berlin: Springer; 1992. p. 3–31.

[33] Mahoney MJ. Human change processes: the scientific foundations of psychotherapy: BasicBooks; 1991.

[34] Kowalik ZJ, Schiepek G, Kumpf K, Roberts LE, Elbert T (1997). Psychotherapy as a chaotic process II. The application of nonlinear analysis methods on quasi time series of the client-therapist interaction: a nonstationary approach. Psychother Res.

[35] Tschacher W, Scheier C, Hashimoto Y (1997). Dynamical analysis of schizophrenia courses. Biol Psychiatry.

[36] Schiepek G, Kowalik ZJ, Schütz A, Köhler M, Richter K, Strunk G (1997). Psychotherapy as a chaotic process I. Coding the client-therapist interaction by means of Sequential Plan Analysis and the search for chaos: a stationary approach. Psychother Res.

[37] Delignières D, Ramdani S, Torre K (2006). Fractal analyses for ‘short’ time series: a re-assessment of classical methods. J Math Psychol.

[38] Myin-Germeys I, Oorschot M, Collip D, Lataster J, Delespaul P, Van Os J (2009). Experience sampling research in psychopathology: opening the black box of daily life. Psychol Med.

[39] Schiepek G, Strunk G (2010). The identification of critical fluctuations and phase transitions in short term and coarse-grained time series-a method for the real-time monitoring of human change processes. Biol Cybern.

[40] Van Orden GC, Holden JG, Turvey MT (2003). Self-organization of cognitive performance. J Exp Psychol Gen..

[41] Bak P, Tang C, Wiesenfeld K (1987). Self-organized criticality: an explanation of the 1/f noise. Phys Rev Lett.

[42] Gilden DL (2001). Cognitive emissions of 1/f noise. Psychol Rev.

[43] Wijnants ML. A review of theoretical perspectives in cognitive science on the presence of 1/f scaling in coordinated physiological and cognitive processes. J Nonlinear Dyn. 2014. 10.1155/2014/962043.

[44] Delignières D, Fortes M, Ninot G (2004). The fractal dynamics of self-esteem and physical self. Nonlinear Dynamics Psychol Life Sci.

[45] Kelty-stephen DG, Wallot S. Multifractality versus ( mono-) fractality as evidence of nonlinear interactions across timescales: disentangling the belief in nonlinearity from the diagnosis of nonlinearity in empirical data. Ecol Psychol. 2017. 10.1080/10407413.2017.1368355.

[46] Molenaar PCM, Sinclair KO, Rovine MJ, Ram N, Corneal SE (2009). Analyzing developmental processes on an individual level using nonstationary time series modeling. Dev Psychol.

[47] Dakos V, Van Nes EH, d'Odorico P, Scheffer M (2012). Robustness of variance and autocorrelation as indicators of critical slowing down. Ecology..

[48] Nelson B, McGorry PD, Wichers M, Wigman JTW, Hartmann JA (2017). Moving from static to dynamic models of the onset of mental disorder. JAMA Psychiatry.

[49] Hufford MR, Witkiewitz K, Shields AL, Kodya S, Caruso JC (2003). Relapse as a nonlinear dynamic system: application to patients with alcohol use disorders. J Abnorm Psychol.

[50] Fartacek C, Schiepek G, Kunrath S, Fartacek R, Plöderl M (2016). Real-time monitoring of non-linear suicidal dynamics: methodology and a demonstrative case report. Front Psychol.

[51] Lutz W, Ehrlich T, Rubel J, Hallwachs N, Röttger M-A, Jorasz C (2013). The ups and downs of psychotherapy: sudden gains and sudden losses identified with session reports. Psychother Res.

[52] Olthof M, Hasselman F, Strunk G, van Rooij M, Aas B, Helmich MA, et al. Critical fluctuations as an early-warning signal for sudden gains and losses in patients receiving psychotherapy for mood disorders. Clin Psychol Sci. 2020;8:25–35.

[53] Helmich MA, Wichers M, Olthof M, Strunk G, Aas B, Aichhorn W (2020). Sudden gains in day-to-day change: revealing nonlinear patterns of individual improvement in depression. J Consult Clin Psychol.

[54] Hayes AM, Laurenceau J-P, Feldman G, Strauss JL, Cardaciotto L (2007). Change is not always linear: the study of nonlinear and discontinuous patterns of change in psychotherapy. Clin Psychol Rev.

[55] Olthof M, Hasselman F, Strunk G, Aas B, Schiepek, G, Lichtwarck-aschoff A. Destabilization in self-ratings of the psychotherapeutic process is associated with better treatment outcome in patients with mood disorders. Psychother Res. 2020;30:520-531. 10.1080/10503307.2019.1633484 31256713

[56] Lichtwarck-Aschoff A, Hasselman F, Cox RFA, Pepler D, Granic I (2012). A characteristic destabilization profile in parent-child interactions associated with treatment efficacy for aggressive children. Nonlinear Dynamics Psychol Life Sci..

[57] Gelo OCG, Salvatore S (2016). A dynamic systems approach to psychotherapy: a meta-theoretical framework for explaining psychotherapy change processes. J Couns Psychol.

[58] Scheffer M, Bascompte J, Brock WA, Brovkin V, Carpenter SR, Dakos V (2009). Early-warning signals for critical transitions. Nature..

[59] Kelso JAS. Self-organizing dynamical systems. In: Smelser NJ, Baltes PB, editors. Int Encycl Soc Behav Sci. Pergamon-Elsevier; 2001. p.13844–13850.

[60] Guastello SJ (2009). Chaos as a psychological construct: historical roots, principal findings, and current growth directions. Nonlinear Dynamics Psychol Life Sci..

[61] Schiepek G, Gelo O, Viol K, Kratzer L, Orsucci F, de Felice G, et al. Complex individual pathways or standard tracks? A data-based discussion on the trajectories of change in psychotherapy. Couns Psychother Res. 2020. 10.1002/capr.12300.

[62] Strunk G, Lichtwarck-Aschoff A (2019). Therapeutic chaos. J Person-Oriented Res.

[63] Kossakowski J, Groot P, Haslbeck J, Borsboom D, Wichers M. Data from ‘critical slowing down as a personalized early warning signal for depression.’ J Open Psychol Data 2017; 10.5334/jopd.29.

[64] Wichers M, Groot PC, Psychosystems, ESM Group, EWS Group. Critical slowing down as a personalized early warning signal for depression. Psychother Psychosom. 2016;85:114–116. 10.1159/000441458 26821231

[65] Buuren S van, Groothuis-Oudshoorn K. MICE: multivariate imputation by chained equations in R. J Stat Softw. 2011; 10.18637/jss.v045.i03.

[66] R Core Team. R: a language and environment for statistical computing. R Found Stat Comput Vienna, Austria. 2017; Available from: https://www.r-project.org/.

[67] Wood SN (2006). Generalized additive models: an introduction with R.

[68] Bastiaansen JA, Kunkels YK, Blaauw F, Boker SM, Ceulemans E, Chen M, et al. Time to get personal? The impact of researchers’ choices on the selection of treatment targets using the experience sampling methodology. PsyArXiv; 2019. Available from: psyarxiv.com/c8vp7. 10.1016/j.jpsychores.2020.110211 PMC8287646 32862062

[69] Bringmann LF, Hamaker EL, Vigo DE, Aubert A, Borsboom D, Tuerlinckx F. Changing dynamics: time-varying autoregressive models using generalized additive modeling. Psychol Methods. 2016. 10.1037/met0000085. 10.1037/met0000085 27668421

[70] Trapletti A, Hornik K. tseries: time series analysis and computational finance. R package version 0.10–47. 2019.

[71] James NA, Matteson DS. ecp: an R package for nonparametric multiple change point analysis of multivariate data. arXiv Prepr arXiv13093295. 2013;.

[72] Sugihara G, May RM (1990). Nonlinear forecasting as a way of distinguishing chaos from measurement error in time series. Nature..

[73] Ye H, Clark A, Deyle E, Sugihara G. rEDM: an R package for empirical dynamic modeling and convergent cross mapping. https://cran.r-project.org/web/packages/rEDM/vignettes/rEDM.html. Accessed 8 Apr 2020.

[74] Takens F, Rand D, Young L-S (1981). Detecting strange attractors in turbulence. Dynamical systems and turbulence, Warwick 1980.

[75] Riley MA, Van Orden GC. Tutorials in contemporary nonlinear methods for the behavioral sciences. http://www.nsf.gov/sbe/bcs/pac/nmbs/nmbs.jsp. Accessed 1 Mar 2005.

[76] Olthof M, Hasselman F, Wijnants M, Lichtwarck-Aschoff A. Psychological dynamics are complex: a comparison of scaling, variance, and dynamic complexity in simulated and observed data. In: Selbstorganisation–ein Paradigma für die Humanwissenschaften. Wiesbaden: Springer; 2020. p. 303–316.

[77] Wijnants ML, Cox R, Hasselman F, Bosman A, Van Orden G (2012). A trade-off study revealing nested timescales of constraint. Front Physiol.

[78] Hasselman F (2015). Classifying acoustic signals into phoneme categories: average and dyslexic readers make use of complex dynamical patterns and multifractal scaling properties of the speech signal. PeerJ..

[79] Ward RM, Kelty-Stephen DG (2018). Bringing the nonlinearity of the movement system to gestural theories of language use: multifractal structure of spoken English supports the compensation for coarticulation in human speech perception. Front Physiol.

[80] Gilden DL, Thornton T, Mallon MW (1995). 1/f noise in human cognition. Science..

[81] Kello CT, Anderson GG, Holden JG, Van Orden GC (2008). The pervasiveness of 1/f scaling in speech reflects the metastable basis of cognition. Cogn Sci.

[82] Kello CT, Brown G, Cancho RF, Holden J, Linkenkaer-Hansen K, Rhodes T (2009). Scaling laws in cognitive science. Proceedings of the Annual Meeting of the Cognitive Science Society.

[83] Kuznetsov N, Wallot S (2011). Effects of accuracy feedback on fractal characteristics of time estimation. Front Integr Neurosci.

[84] Van Orden GC, Holden JG, Turvey MT (2005). Human cognition and 1/f scaling. J Exp Psychol Gen.

[85] Rigoli LM, Holman D, Spivey MJ, Kello CT. Spectral convergence in tapping and physiological fluctuations: coupling and independence of 1/f noise in the central and autonomic nervous systems. Front Hum Neurosci. 2014. 10.3389/fnhum.2014.00713. 10.3389/fnhum.2014.00713 PMC4160925 25309389

[86] Wijnants ML, Bosman AMT, Hasselman FW, Cox RFA, Van Orden GC (2009). 1/f scaling in movement time changes with practice in precision. Nonlinear Dynamics Psychol Life Sci..

[87] Ramachandran B. On the “strong memorylessness property” of the exponential and geometric probability laws. Sankhyā Indian J Stat Ser A. 1979:244–51.

[88] Rubel JA, Zilcha-mano S, Giesemann J, Prinz J, Lutz W. Predicting personalized process-outcome associations in psychotherapy using machine learning approaches — a demonstration. Psychother Res. 2019. 10.1080/10503307.2019.1597994. 10.1080/10503307.2019.1597994 30913982

[89] Hasselman F, Bosman AMT. Studying complex adaptive systems with internal states: a recurrence network approach to the analysis of multivariate time series data representing self-reports of human experience. Front Appl Math Stat. 2020;6:9.

[90] Olthof M, Hasselman F, Oude Maatman F, Bosman AMT, Lichtwarck-Aschoff A. Adaptive DynAmic Pattern Theory (ADAPT) of Psychopathology. 2020. https://psyarxiv.com/f68ej/. 10.1037/abn0000740 37126062

[91] Hayes AM, Andrews LA (2020). A complex systems approach to the study of change in psychotherapy. BMC Med.

[92] Beran J (1992). Statistical methods for data with long-range dependence. Stat Sci.

[93] Diniz A, Wijnants ML, Torre K, Barreiros J, Crato N, Bosman AMT, et al. Human Movement Science Contemporary theories of 1/f noise in motor control. Hum Mov Sci 2011;30:889–905. 10.1016/j.humov.2010.07.006 21196059

[94] Hasselman F. When the blind curve is finite: dimension estimation and model inference based on empirical waveforms. Front Physiol. 2013. 10.3389/fphys.2013.00075. 10.3389/fphys.2013.00075 PMC3619109 23580349

[95] Wagenmakers E, Farrel S, Ratcliff R (2004). Estimation and interpretation of 1/f a noise in human cognition. Psychon Bull Rev.

[96] Van Orden GC, Kloos H, Wallot S, Hooker C (2011). Living in the pink. Intentionality, wellbeing, and complexity. Handbook of the philosphy of science.

[97] Torre K, Delignieres D, Lemoine L (2007). Detection of long-range dependence and estimation of fractal exponents through ARFIMA modeling. Br J Math Stat Psychol.

[98] Haslbeck JMB, Ryan O, Robinaugh D, Waldorp L, Borsboom D. Modeling psychopathology: from data models to formal theories 2019. 10.31234/osf.io/jgm7f. 10.1037/met0000303 PMC10259162 34735175

[99] Granger CWJ (1969). Investigating causal relations by econometric models and cross-spectral methods author. Econometrica..

[100] Sugihara G, May R, Ye H, Hsieh C, Deyle E, Fogarty M (2012). Detecting causality in complex ecosystems. Science..

[101] Haslbeck J, Ryan O. Recovering bistable systems from psychological time series. 2019; 10.31234/osf.io/kcv3s.

[102] Heino MTJ, Knittle KP, Noone C, Hasselman F, Hankonen N. Studying behaviour change mechanisms under complexity; 2020. 10.31234/osf.io/fxgw4. 10.3390/bs11050077 PMC8156531 34068961

[103] Pham T (2020). The recurrence dynamics of personalized depression. In: Proceedings of the Australasian computer science week multiconference.

[104] Scheffer M, Carpenter SR, Lenton TM, Bascompte J, Brock W, Dakos V (2012). Anticipating critical transitions. Science..

[105] Granic I (2005). Timing is everything: developmental psychopathology from a dynamic systems perspective. Dev Rev.

[106] Thelen E, Smith LB (1994). A dynamic systems approach to the development of cognition and action.

[107] Schiepek G, Stöger-Schmidinger B, Aichhorn W, Schöller H, Aas B. Systemic case formulation, individualized process monitoring, and state dynamics in a case of dissociative identity disorder. Front Psychol. 2016. 10.3389/fpsyg.2016.01545. 10.3389/fpsyg.2016.01545 PMC5072376 27812338

[108] Smit AC, Snippe E, Wichers M (2019). Increase in depressive symptoms more than 2 months before it happens in individual patients. Psychother Psychosom.

[109] Burger J, van der Veen DC, Robinaugh D, Quax R, Riese H, Schoevers RA, et al. Bridging the gap between complexity science and clinical practice by formalizing idiographic theories: a computational model of functional analysis. BMC Med. 2020;18:99. 10.1186/s12916-020-01558-1 PMC7333286 32264914

[110] Fisher AJ, Soyster PD. Generating Accurate Personalized Predictions of Future Behavior: A Smoking Exemplar. 10.31234/osf.io/e24v6.

[111] Brunton SL, Proctor JL, Kutz JN (2016). Discovering governing equations from data by sparse identification of nonlinear dynamical systems. Proc Natl Acad Sci.

[112] Stiles WB, Shapiro DA (1994). Disabuse of the drug metaphor: psychotherapy process-outcome correlations. J Consult Clin Psychol.

[113] Schiepek G, Aichhorn W, Gruber M, Strunk G, Bachler E, Aas B (2016). Real-time monitoring of psychotherapeutic processes: concept and compliance. Front Psychol.

